# Factors affecting the mature use of electronic medical records by primary care physicians: a systematic review

**DOI:** 10.1186/s12911-021-01434-9

**Published:** 2021-02-19

**Authors:** Rana Melissa Rahal, Jay Mercer, Craig Kuziemsky, Sanni Yaya

**Affiliations:** 1 Population Health Program, University of Ottawa, 25 University Private, Ottawa, Ontario K1N 7K4 Canada; 2 Bruyère Continuing Care, Ottawa, Ontario Canada; 3 Office of Research Services, MacEwan University, Edmonton, Alberta Canada; 4 School of International Development and Global Studies, University of Ottawa, Ottawa, Ontario Canada; 5 The George Institute for Global Health, University College London, London, UK

**Keywords:** Electronic health records, Primary health care, General practitioners

## Abstract

**Background:**

Despite a substantial increase in the adoption of electronic medical records (EMRs) in primary health care settings, the use of advanced EMR features is limited. Several studies have identified both barriers and facilitating factors that influence primary care physicians’ (PCPs) use of advanced EMR features and the maturation of their EMR use. The purpose of this study is to explore and identify the factors that impact PCPs’ mature use of EMRs.

**Methods:**

A systematic review was conducted in accordance with the Cochrane Handbook. The MEDLINE, Embase, and PsycINFO electronic databases were searched from 1946 to June 13, 2019. Two independent reviewers screened the studies for eligibility; to be included, studies had to address factors influencing PCPs’ mature use of EMRs. A narrative synthesis was conducted to collate study findings and to report on patterns identified across studies. The quality of the studies was also appraised.

**Results:**

Of the 1893 studies identified, 14 were included in this study. Reported factors that influenced PCPs’ mature use of EMRs fell into one of the following 5 categories: technology, people, organization, resources, and policy. Concerns about the EMR system’s functionality, lack of physician awareness of EMR functionality, limited physician availability to learn more about EMRs, the habitual use of successfully completing clinical tasks using only basic EMR features, business-oriented organizational objectives, lack of vendor training, limited resource availability, and lack of physician readiness were reported as barriers to PCPs’ mature use of EMRs. The motivation of physicians, user satisfaction, coaching and peer mentoring, EMR experience, gender, physician perception, transition planning for changes in roles and work processes, team-based care, adequate technical support and training, sharing resources, practices affiliated with an integrated delivery system, financial incentives, and policies to increase EMR use all had a favorable impact on PCPs’ use of advanced EMR features.

**Conclusions:**

By using a narrative synthesis to synthesize the evidence, we identified interrelated factors influencing the mature use of EMRs by PCPs. The findings underline the need to provide adequate training and policies that facilitate the mature use of EMRs by PCPs.

*Trial registration*: PROSPERO CRD42019137526.

## Background

As the population ages, the prevalence of chronic disease increases, and primary health care needs are becoming increasingly complex to support [1]. As a result, there is a need to redesign primary care to improve the quality of health care services to effectively support the health of the population while also addressing issues such as rising costs [2]. Policymakers have developed system efficiencies to meet the demands of primary health care and Electronic medical records (EMRs) are one health information system recommended to facilitate point of care delivery by primary health care professionals [3, 4].

Although the adoption of EMRs has increased internationally [3], the use of advanced EMR features has been limited, particularly in Canada [5]. However, there is evidence of modest improvement in the use of advanced EMR features, such as electronic reminders prompting follow-up for preventive care and clinical decision support tools in preventative care and disease management [6, 7].

“Meaningful use” is a term from the Health Information Technology for Economic and Clinical Health (HITECH) Act passed in the United States [8]. The HITECH act defined “meaningful use” as the use of certified EMR technology in a meaningful manner (e.g., electronic prescribing) to ensure that the certified EMR improved the quality of care [8]. As such, clinicians using certified EMRs must report information on the quality of care, and other measures and specific objectives were set out for clinicians to mature in their use of EMR features [9]. In Canada, “meaningful use” has been used to study mature use of EMRs [10]. In the province of Ontario, OntarioMD, a cooperative owned by the Ontario Medical Association and funded by the provincial government, certifies EMRs and aims to increase the mature use of EMRs in the province [11]. OntarioMD developed the EMR Maturity Model [12] to assist clinicians in the mature use of EMRs. The model is designed so that clinicians can measure their level of EMR use for a certain process (e.g., prevention and screening) across 6 levels of EMR maturity (0 [paper-based] to 5 [integrated]). It is intended to help clinicians identify their current EMR use level to determine how to help them mature in their EMR use [12]. Likewise, this study defines maturity as the maturation of the user’s skill set and clinical processes in using a health information system, rather than the maturity of a product itself (i.e., type of features implemented in an EMR) [13].

Although there are a myriad of factors that have been found to impact EMR adoption and implementation [6, 14, 15], to date, there has been no systematic review exploring the factors impacting PCPs’ mature use of EMRs. Studies have highlighted the problem of a tiered EMR ceiling effect [16–18], which occurs when a user is not yet an EMR expert and barriers constrain him or her from learning more advanced EMR features and reaching maturity [18]. Furthermore, providing support to enable maturity in EMR use has been identified as a Canadian research priority [19]. Therefore, there is an opportunity and a critical need to examine how to support PCPs in their use of the advanced features of their EMRs. It is important to identify factors that prevent PCPs from using the full potential of EMRs to enable them to support greater clinical value and quality healthcare service delivery.

## Methods

In accordance with the Cochrane Handbook [20], a systematic review was conducted based on the Preferred Reporting Items for Systematic Reviews and Meta-Analyses (PRISMA) guidelines [21]. On September 20, 2019, the protocol for this systematic review was registered with the International Prospective Register of Systematic Reviews (PROSPERO), and it was last updated on November 30, 2020 (registration number CRD42019137526).

### Data sources and search strategy

An information specialist and the primary author (RR) designed the search strategy. The search strategy covered 3 core search terms: (1) PCPs, (2) electronic medical records (EMRs), and (3) maturity. Medical subject headings (MeSH) and thesaurus terms related to the above 3 core search terms were used for the literature search strategies. Three electronic databases were searched, with no language restriction: MEDLINE, PsycINFO, and Embase. The PROSPERO registry was also searched for ongoing or recently completed relevant systematic reviews.

**Fig. 1:**
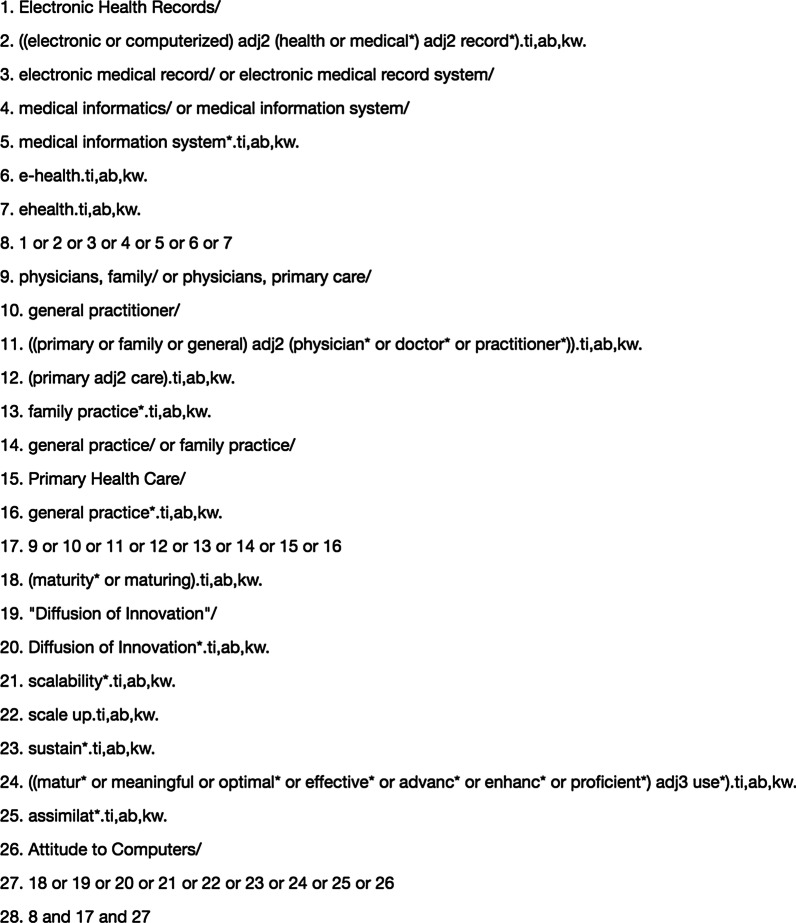
Ovid MEDLINE database search strategy

Other sources of studies were also investigated: Google Scholar was searched using topic keywords (EMR, PCP, maturity), reference lists of eligible studies were manually searched, citation analysis using the database Scopus was conducted on key studies, and key journals in the field (International Journal of Medical Informatics, Journal of the American Medical Informatics Association, Journal of Medical Internet Research) were searched for relevant studies. In addition, the gray literature of theses (ProQuest Dissertations and Thesis Global) was searched. The search strategy included studies published from 1946 to June 13, 2019 (see Fig. 1). Furthermore, the search strategy (shown in Fig. 1) included studies written in any language to not exclude studies where the abstracts were not written in English but the full texts were in English. However, during full text screening, studies not written in English were excluded due to limited resources to translate the study into English.

### Inclusion criteria

As described previously, this review followed the OntarioMD EMR Maturity Model’s definition of maturity [12]. As such, the primary outcome is the identification of factors impacting PCPs’ mature use of EMRs. Study inclusion and exclusion criteria were created to guide the identification and selection of studies eligible for the systematic review (see Table 1).

**Table 1:** Study inclusion criteria

	Inclusion criteria
Type of study	Study designs must report original primary data that answer the question “what factors affect the mature use of EMRs by PCPs?”
Type of participants	The primary user of the EMR must be a PCP For this review, PCPs are medically trained physicians who deliver primary health care PCPs include general practitioners, family doctors, primary healthcare doctors, family physicians, and family practitioners
Intervention	The intervention in question is the mature use of EMRs by PCPs to support primary care delivery. This review uses Canada Health Infoway’s definition of an EMR: “A computer-based patient record specific to a single clinical practice” [22]
Setting	Any primary health care setting This review followed the Institute of Medicine’s definition of primary health care: “The provision of integrated, accessible health care services by clinicians who are accountable for addressing a large majority of personal health care needs, developing a sustained partnership with patients and practicing in the context of family and community” [23]
Language	Studies where the full text is written in English

### Data selection and extraction

Search results were imported into Covidence (a web-based software platform that supports the development of systematic reviews) [24] which allowed us to remove duplicate studies. Two independent reviewers conducted the first level of screening of the literature, using citation titles and abstracts only to identify potentially relevant studies that met the inclusion criteria (see Table 1). Both reviewers had to agree on the ineligibility of a citation for it to be excluded. Finally, 2 independent reviewers screened the full texts of eligible studies to determine the final set of studies to be included in this review.

The 2 reviewers independently extracted data; any disagreements were resolved through consensus. A structured data collection tool developed from a standardized data extraction form by the Cochrane library [25] was used to ensure the systematic extraction of data, as the extracted data were used to synthesize the findings. The data collection tool was pilot tested on 5 randomly selected eligible studies. The structured form guided the extraction of key study characteristics, including country, design, number of participants, data collection procedures, and barriers and facilitating factors influencing PCPs’ mature use of EMRs.

### Quality appraisals

The quality of eligible studies was assessed by the 2 reviewers using the Public Health Ontario meta-tool for quality appraisal for public health evidence (PHO MetaQAT) [26]. Due to the heterogeneity of the design of the eligible studies, the PHO MetaQAT was used because it was developed to appraise diverse study designs (including research published as gray literature) while ensuring a high degree of rigor. The reviewers independently rated each study based on its (1) relevancy (the study met inclusion and exclusion criteria), (2) reliability (there was sufficient reporting on the conduct of the study), (3) validity (measures were used to decrease the likelihood of errors or bias, and results could be generalized to a wider population), and (4) applicability (the evidence could be applied to public health practice). Any disagreements were resolved by discussion. Authors of the included studies were contacted, as needed, to obtain further information or clarify any questions about a study. Unlike many quality appraisal tools, the PHO MetaQAT does not use numeric scoring to appraise quality since numerical summary scores mask important details [26]. Instead, the PHO MetaQAT is designed to document all important details to provide transparency.

### Data analysis

Narrative synthesis was chosen as the method for synthesis rather than a statistical meta-analysis or other forms of synthesis because of resource limitations and time constraints. A qualitative narrative synthesis primarily relies on the use of text to summarize and explain the findings of the synthesis [27]. Narrative synthesis is useful in synthesizing evidence of different types (qualitative, quantitative, etc.) and useful in comparing similarities and differences across studies [28]. Findings were synthesized iteratively following the guidelines established by Popay et al. [27] for conducting a narrative synthesis. Popay et al. [27] recommend a generic framework that offers various tools and techniques to guide the process of narrative synthesis. The tools and techniques suggested by Popay et al. [27] helped to increase the transparency of the qualitative narrative synthesis process and the reliability of the findings and conclusions of this review.

### Exploring relationships in the data

Textual descriptions are one technique recommended by Popay et al. [27] to compare and contrast findings across studies. A review finding is an analytic output (e.g., a theme, category, theory) from a qualitative evidence synthesis that, based on data from primary studies, describes a phenomenon that covers the intent of the review question [29]. For the synthesis of review findings, textual descriptions were performed for each study included to explore reported factors that influence PCPs’ mature use of EMRs and identify relationships within and among studies. Thematic analysis was used to organize and summarize findings in a concise way from a large body of evidence [27]. Text on the factors that impact PCPs’ mature use of EMRs was extracted from the studies and entered into a Microsoft Excel spreadsheet. Extracted data were independently read through thoroughly by RR to inductively code and identify the salient themes (factors) for PCPs’ mature use of EMRs [30]. Findings were reported in textual format under the major themes, ensuring that data were reported in a structured and organized fashion [27].

### Assessment of confidence in the synthesis findings

Assessing the robustness of the narrative synthesis allowed us to evaluate the strength of the evidence for drawing conclusions about the facilitators and/or barriers to PCPs’ mature use of EMRs identified in the synthesis [27]. The GRADE-Confidence in the Evidence from Reviews of Qualitative Research (CERQual) approach was used to assess each qualitative review finding [31]. The method has been helpful for decision makers and policy designers who use qualitative evidence to inform policies and interventions about various topics, such as healthcare [29]. The GRADE-CERQual approach uses 4 components to assess confidence in review findings: (1) the methodological limitations of the studies included [32], (2) the coherence and fit between data from primary studies and the review findings [33], (3) the adequacy of data, degree of richness, and quantity of data supporting a review finding [34], and (4) the relevance of the studies included in terms of whether they reflect the context determined by the review question [35]. Furthermore, as per PRISMA guidelines [21], the characteristics of eligible studies are presented in a narrative format. The MeaSurement Tool to Assess Review (AMSTAR) was also used to evaluate the methodological quality of this systematic review [36].

## Results

### Characteristics of the studies included

**Fig. 2:**
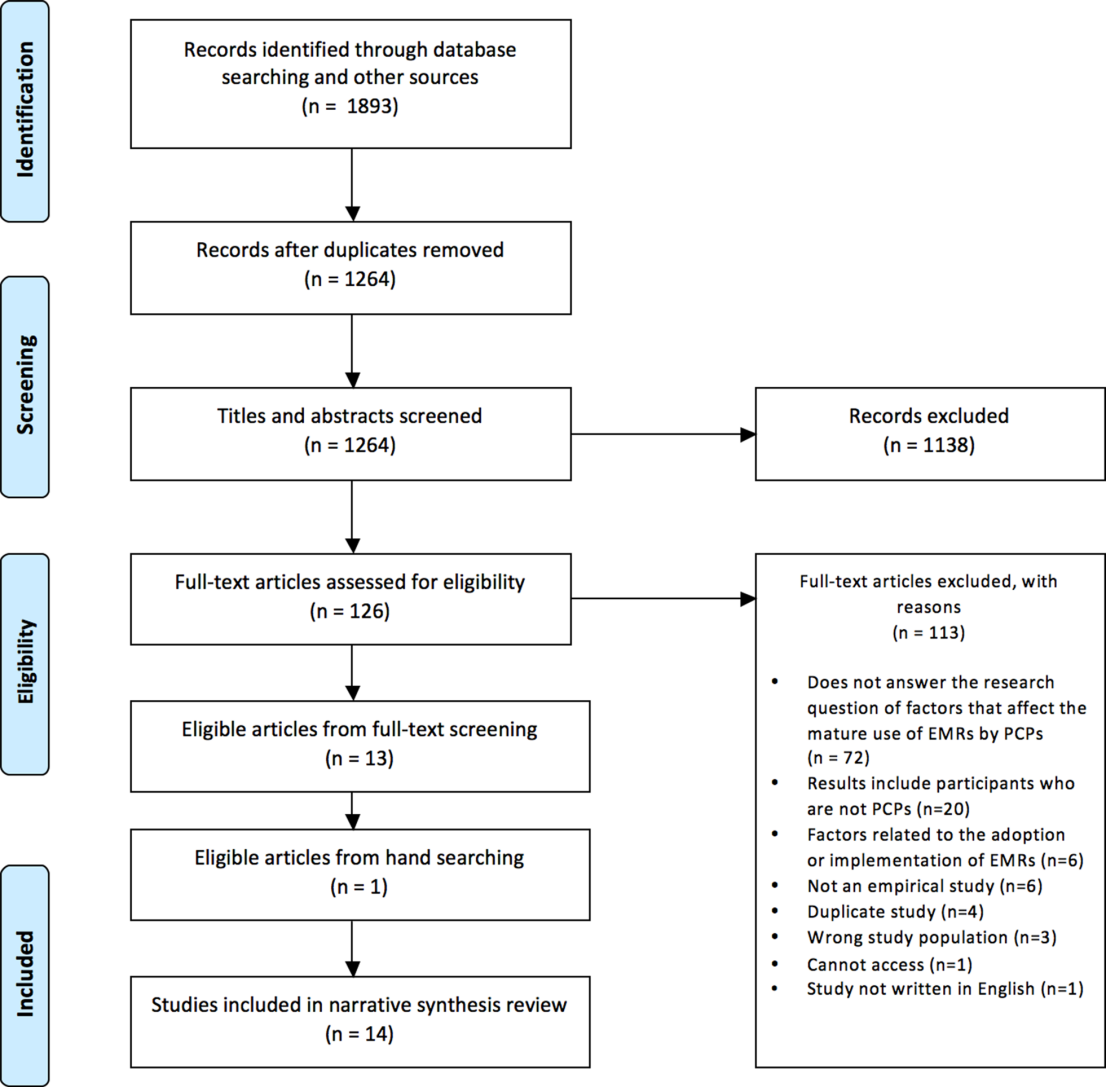
PRISMA flowchart showing the selection process of eligible studies included in the review

Of the 1264 studies screened, 14 studies were included (see Fig. 2): 5 were conducted in the United States [7, 37–40], 7 were conducted in Canada [10, 16, 18, 41–44], 1 was conducted in Israel [45], and 1 was an international study [3] conducted in 10 countries, including Australia, Canada, France, Germany, the Netherlands, New Zealand, Norway, Switzerland, the United Kingdom, and the United States. The studies were methodologically diverse, including 4 qualitative studies [7, 18, 44, 45], 6 cross-sectional studies [3, 37, 39, 41–43], 3 mixed-methods studies [10, 16, 38], and 1 quantitative descriptive study [40]. These studies were published between 2009 and 2019 (see Table 2 for full study details).

**Table 2:** Characteristics of eligible studies

Author (year), country	Design	Sample size of PCPs	Data collection
Audet et al. (2014), US [39]	Cross-sectional study	1012	Self-administered, structured questionnaire
Clarke et al. (2016), US [38]	Mixed-model approach combining qualitative and quantitative research methods	16	Video analyses Qualitative participant debriefing sessions (narrative feedback) Quantitative performance measures (percent task success, time-on-task, mouse clicks, and mouse movements) System usability scale (a survey instrument that allows physicians to rank the system’s usability)
DesRoches et al. (2013), US [37]	Cross-sectional study	1164	Self-administered, structured questionnaire
Goetz et al. (2012), US [7]	Qualitative methods	14	Telephone interviews On-site visits involving interviews and observation National Survey of Physician Organizations
Jones et al. (2018), Canada [44]	Qualitative methods, observational study	6	Semistructured interviews using the EMR Progress Assessment survey tool Observations of data quality in EMRs and of physician’s workflow
Lynch et al. (2014), US [40]	Quantitative methods, descriptive study	101,584	From 10 databases: Administrative data from Regional Extension Center program’s customer relationship management database merged with 9 other secondary data source databases
Paré et al. (2015), Canada [41]	Cross-sectional study	331	Self-administered structured questionnaire
Randhawa et al. (2018), Canada [10]	Mixed-model approach combining qualitative and quantitative research methods	18	Survey, questionnaire Follow-up interviews
Raymond et al. (2015), Canada [42]	Cross-sectional study	331	Self-administered structured questionnaire
Raymond et al. (2019), Canada [43]	Cross-sectional study	331	Self-administered structured questionnaire
Schoen et al. (2012), 10 countries (Australia, Canada, France, Germany, Netherlands, New Zealand, Norway, Switzerland, UK, US) [3]	Cross-sectional study	Sample sizes of PCPs ranged from 500 to more than 2000 for the 10 countries	Phone interviews Mail surveys using a questionnaire
Shachak et al. (2009), Israel [45]	Qualitative methods	25	Semistructured interviews Field observations
Trudel et al. (2017), Canada [18]	Qualitative methods	15	Semistructured interviews Field notes from observations Consultation of relevant documents (e.g., user manuals)
Watt (2014), Canada [16]	Mixed-model approach combining qualitative and quantitative research methods	29	Questionnaire, survey Notes from action plans developed with coach and physician Clinical Value Model (CVM) assessment tool (86 questions, subjective assessment tool administered by the coach) CVM feedback survey (web-based survey for physicians to complete) Notes from practice optimization plan

### Factors that impact PCPs’ mature use of EMRs

**Fig. 3:**
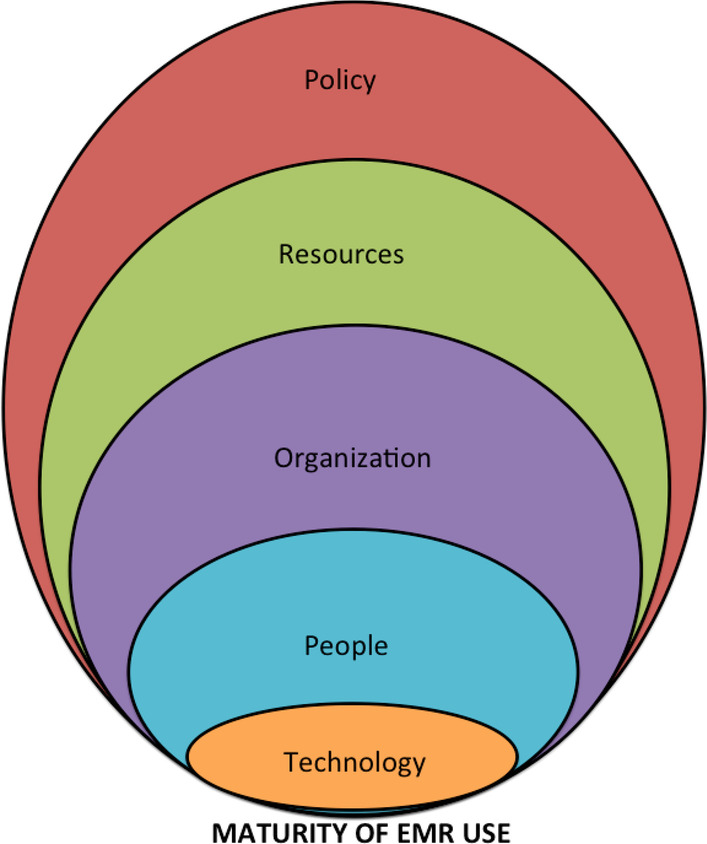
Conceptual model of influential factors in PCPs’ mature use of EMRs

The salient factors were found to be best described by 5 overarching themes: technology, people, organization, resources, and policy. The conceptual model shown below (Fig. 3) portrays how each of these factors is a lens to look at PCPs’ mature use of EMRs. Moreover, the CERQual approach uses 4 levels to describe the overall assessment of confidence in a review finding: high, moderate, low, or very low [29]. A summary of the review findings and the CERQual assessments is shown in Table 3. (See Additional file 1 for overall confidence assessments and descriptions for confidence assessments for each finding.)

**Table 3:** Summary of narrative synthesis findings

Review findings (subthemes and summaries)	Contributing studies	CERqual confidence in the evidence
**Technology**		
**EMR system's functionality** – user-friendliness of EMR system, ease of use, and the comprehensiveness of clinical functionalities that fit the main medical tasks of PCPs impacted their use of advanced EMR features	Shachak et al. [45], Raymond et al. [43], Paré et al. [41], Goetz et al. [7]	Moderate confidence
**People**		
**Physician characteristics** – gender was found to have an impact on the mature use of EMRs; advanced EMR features were more often used by female physicians	Randhawa et al. [10], Paré et al. [41]	Low confidence
**EMR experience** – use of advanced EMR features increased most among female PCPs who had the least EMR experience	Randhawa et al. [10]	Very low confidence
**Physician perception** – PCPs who perceived that the use of advanced EMR features would have a positive effect on their individual performance (e.g., communication, interaction with patients and other care providers) and their clinic’s performance (e.g., quality of care and patient safety) used advanced EMR features	Paré et al. [41], Shachak et al. [45]	Low confidence
**Awareness of EMR functionality** – PCPs’ lack of awareness of all the available advanced EMR features could be a barrier to the maturing of their use of EMRs	Watt [16], Trudel et al. [18], Goetz et al. [7]	Moderate confidence
**Physician readiness** – lack of physician readiness to use advanced EMR features even though they had the capability could be a barrier to their mature use of EMRs	Watt [16], Trudel et al. [18]	Low confidence
**Physician motivation** – PCPs’ motivation to improve and become proficient in using EMRs was found to facilitate their mature use of EMRs. Whereas PCPs that were not motivated continued to use basic functions and consciously ignored advanced EMR features	Jones et al. [44], Trudel et al. [18]	Moderate confidence
**User satisfaction** – EMR user satisfaction could facilitate the mature use of EMRs	Watt [16], Raymond et al. [42]	Moderate confidence
**Physician availability** – inadequate time to learn more advanced EMR features and to invest in continuous learning to better use them was found to prevent mature use by PCPs	Trudel et al. [18], Goetz et al. [7]	Low confidence
**Habitual use** – successful performance of clinical tasks with only basic use of EMRs was found to be a barrier to mature use of EMRs	Trudel et al. [18]	Very low confidence
**Patient concerns** – the impersonality of EMR data entry during medical exams led to physicians’ dissatisfaction with and resistance to using advanced EMR features	Goetz et al. [7]	Very low confidence
**Organization**		
**Practice type** – advanced EMR users have been shown to be affiliated in a practice with an integrated delivery system where PCPs collaborate closely with other health and social services professionals, that shares resources, and that is eligible for financial incentives	Audet et al. [39], Paré et al. [41], DesRoches et al. [37]	Moderate confidence
**Practice size** – PCPs in larger practices (5 or more full-time-equivalent PCPs) were more likely to be advanced EMR users compared to PCPs from smaller practices (less than 2 full-time PCPs)	Audet et al. [39], Schoen et al. [3], Goetz et al. [7]	Moderate confidence
**Organizational objectives** – clinical objectives to use EMRs on a daily basis and integrate them into the organization were absent or secondary once a clinic’s operational objectives for EMR use had been met. In addition to a business-oriented motivation, thwarted any effort to extend EMR use was found to be a barrier to the mature use of EMRs	Trudel et al. [18]	Very low confidence
**Team-based care** – team-based methods such as assigning responsibility to nurses or other staff to enter patient data into EMRs or retrieve it allowed physicians to focus on patient care and facilitated the use of advanced EMR features	Goetz et al. [7]	Very low confidence
**Transition planning** – planning for changes in roles and responsibilities, redesigning work processes, and developing up-to-date policies and procedures in a practice when implementing advanced EMR features facilitated their advanced use	Goetz et al. [7]	Very low confidence
**Resources**		
**Vendor training** – limited and poor quality vendor training such as short training sessions and material based on theory rather than clinical practice, biased physicians towards using only basic EMR features	Watt [16], Trudel et al. [18]	Low confidence
**Training** – adequate training (e.g., video training, training focused on clinical benefits, group training, procedural work flow manuals, 1-on-1 guidance) increased PCPs’ use of advanced EMR features	Randhawa et al. [10], Goetz et al. [7]	Low confidence
**Coaching and peer mentoring** – coaching by consultants and peer mentoring increased PCPs’ mature use of EMRs	Lynch et al. [40], Watt [16], Jones et al. [44]	Moderate confidence
**Sharing resources** – sharing technical assistance was found to be associated with multifunctional health information technology capacity, electronically exchanging patient information, and electronic patient access	Audet et al. [39], Goetz et al. [7]	Very low confidence
**Financial incentives** – PCPs that received or were eligible for financial incentives were more likely to be able to use advanced EMR features (e.g., electronically exchanging patient information with physicians outside of their practice)	Audet et al. [39], Goetz et al. [7]	Very low confidence
**Technical support** – adequate technical assistance, such as EMR vendors, a health information technology department, or an in-house EMR “go-to person” who supported the configuring of new EMR features and training staff, was a critical factor in the use of advanced EMR features	Goetz et al. [7]	Very low confidence
**Resource availability** – limited sources of information about EMRs was found to prevent their mature use	Trudel et al. [18]	Very low confidence
**Policy**		
**Policies to increase EMR use** – PCPs from small practices in countries that had collaborative and regional policies to increase the spread and use of health information technology were shown to have multifunctional capacity	Schoen et al. [3]	Low confidence

#### Technology

In 4 studies [7, 41, 43, 45], EMR system factors impacted the extent to which physicians used advanced EMR features. In [45], the automaticity of advanced EMR features was found to impede the use of advanced features. This study found that physicians perceived that automaticity in advanced EMR features resulted in errors, where participants (> 60%) reported errors such as typos, adding information to the wrong patient chart, and unintentionally selecting an erroneous item (diagnosis or medication) from a scroll-down list [45]. Additionally, participants perceived that the use of predefined templates negatively impacted patient safety, and they preferred typing over using this advanced feature [45]. Furthermore, a study [7] found that the complexity of EMR features led to physicians having trouble understanding how to use EMR functions and how to incorporate these functions into their work routines. In other studies [41, 43, 45], the EMR system’s user-friendliness and ease of use were important for fuller usage of the system by family physicians. A study [43] found that EMR systems that had comprehensive clinical functionalities (e.g., providing prescriptions electronically) were used more extensively because physicians saw such features as more useful because these clinical functionalities better supported the main clinical tasks they undertook in primary care settings.

#### People

A range of individual factors impacting the mature use of EMRs were raised in 8 studies [7, 10, 16, 18, 41, 42, 44, 45].

### Physician characteristics

Two studies [10, 41] reported that gender impacted the mature use of EMRs. One of these studies [10] found that the use of EMR features for diabetes care increased most among female PCPs, aged 35–44 with low EMR skills and with the least EMR experience, during the duration of the study [10]. The other study [41] found that the majority of advanced users (those who used 21 out of 24 EMR functions) were female physicians. Furthermore, [41] found that advanced users did not differ significantly from basic users (those who used 11 out of 24 EMR functions on average) in terms of their age and medical experience.

### EMR experience

One study [10] found that the use of EMR features for diabetes care increased most among female participants with less EMR experience. This was in contrast to another study that found that advanced users did not differ significantly from basic users in terms of their EMR usage experience [41]. Likewise, in another study [38], no association between extent of EMR experience and proficiency of EMR use was found. However, this study did find that as physicians’ EMR experience and familiarity with the system increased as they became more time efficient in task completion.

### Physician perception

Two studies [41, 45] identified physician perception as playing a role in their mature use of EMR systems. One study found that advanced users, compared to basic users, perceived their EMR system to be significantly easier to use with respect to their interactions with patients and other care providers [41]. This study also found that physicians who perceived that neither individual nor organizational performance improvements were due to their use of an EMR system had a more limited understanding of their EMR system’s functionality, specifically clinical functionalities, compared to physicians who perceived EMR usage as having a positive influence overall on both their individual performance and their clinic’s performance [41]. One other study [45] found that physicians perceived that advanced EMR features (i.e., clinical decision-making aids and alerts of potential adverse drug interactions) reduced their cognitive load, improved communication, improved the quality of care, and enhanced patient safety.

### Awareness of EMR functionality

Three studies [7, 16, 18] revealed that a number of physicians were not aware of all the features that were available in their EMRs, specifically advanced features (e.g., e-referral function). This lack of knowledge could be a barrier to increased mature use of EMR.

### Physician readiness

Two studies [16, 18] found that a lack of physician readiness was a barrier to the use of advanced EMR features. In one study [16], a lack of readiness was referred to as physicians not wanting to consider advanced features even though the EMR had the capability to do so. The other study [18] used the term “organizational inertia,” which also suggests a lack of readiness that was defined as the tendency to be satisfied with the status quo and the outcomes of basic use while consciously ignoring more advanced EMR functionalities.

#### Physician motivation

One study [44] found that physicians’ motivation to improve their use of EMRs was a critical factor that led to success in achieving EMR proficiency. Another study [18] found that when PCPs were not motivated to use the EMR system, they showed a tendency to be satisfied with the status quo and with the outcomes of basic use, and more advanced EMR functionalities were consciously ignored.

#### User satisfaction

Two studies [16, 42] found user satisfaction to be a critical component of EMR use. One of the studies [42] determined that EMR user satisfaction was positively and significantly associated with extended EMR use.

#### Physician availability

Two studies [7, 18] found that having inadequate time to learn more about EMRs inhibited PCPs’ mature use of EMRs. One of the studies [18] found that a decrease in PCPs’ free time for exploring the EMR and having limited time to invest in continuous learning to better use the features affected their mature use of EMRs. Likewise, some PCPs in the other study [7] reported spending weekends learning new EMR functions, and others expressed reluctance to incorporate additional duties into their busy schedules.

#### Habitual use

One study [18] also found that physicians could successfully perform their clinical tasks with minimal use of EMRs. This allowed users to ignore potential (IT-based) alternatives and persist in habitual use that had proven to be satisfactory, efficient, and comfortable.

#### Patient concerns

One study [7] found that some physicians were reluctant to use EMRs because patients were concerned about the impersonal nature of EMR data entry during their medical exam. This resulted in physicians’ resistance to moving forward with advanced EMR functions.

##### Organization

Seven studies addressed several organizational factors that impacted PCPs’ mature use of EMRs [3, 7, 18, 37, 39–41].

### Practice type

Three studies [37, 39, 41] found that practice type was associated with the mature use of EMRs. In one study [41], a significant proportion of advanced users were found to practice in clinics affiliated with a group of family doctors who worked together and in close collaboration with other health and social services professionals. Similarly, in another study [39], physicians who were part of an integrated delivery system that had formal arrangements with other practices to share resources or who were eligible for financial incentives were more likely to demonstrate mature EMR use (e.g., multifunctional health information technology capacity, electronic information exchange with other providers, offering patients electronic access to information, appointments, and prescription refills) than physicians without these incentives.

### Practice size

In 5 studies [3, 7, 37, 39, 40], practice size was associated with physicians’ mature EMR use. In one of these studies [39], practice size was a major determinant of physicians’ mature EMR use (e.g., exchanging patient information electronically and providing electronic access to their patients), and there was a fourfold difference between solo and large practices in achieving multifunctional health information technology capacity (11% vs. 45%). Another study [7] reported that advanced EMR use was more often found in larger practices because of the availability of technical and administrative support. An international study [3] found that Australian, Canadian, New Zealand, Swiss, and US practices with 5 or more full-time-equivalent physicians were significantly more likely to have multifunctional capacity (i.e., using an EMR and at least two electronic functions in the following domains: the generation of patient information, the generation of a patient registry and panel information, order entry management, decision support) than practices with fewer than 2 full-time-equivalent physicians. In [40], physicians in small primary care practices were found to make robust progress towards meaningful EMR use. In [41], advanced users did not differ significantly from basic users in terms of their practice size and location (urban vs. rural).

### Organizational objectives

One study [18] found that the absence or secondary nature of a clinic’s EMR assimilation phase (i.e., when the EMR was used on a daily basis and integrated into the organization) when a clinic’s operational objectives had been met by the EMR system, was an important factor that prevented mature use. The same study [18] also found that primary health care clinics that wanted to use the EMR to address business-related issues (e.g., operational efficiency) thwarted any effort to extend EMR use to reap more benefits.

### Team-based care

Study [7] found that team-based methods such as giving responsibility to nurses or other team members to collect and enter most patient information into the EMRs facilitated the use of advanced EMR features because they allowed physicians to focus on patient care.

#### Transition planning

One study [7] found that planning for changes in roles and responsibilities, redesigning work processes, and developing up-to-date policies and procedures when implementing advanced EMR features facilitated the advanced use of EMR features in practices. The study also found that practices that did not proactively redesign work processes around new advanced EMR features resulted in physicians’ limited use or nonuse of these advanced features because they lacked an understanding of the rationale for advanced feature use.

#### Resources

Seven studies highlighted the importance of resources as a factor that influenced PCPs’ mature use of EMRs [7, 10, 16, 18, 39, 40, 44].

### Training

In 4 studies, training impacted physicians’ mature use of EMRs [7, 10, 16, 18]. One study [10] found that video tutorial training resulted in an increase in PCPs’ use of advanced EMR features for diabetes care. The study also found that the use of EMR features for diabetes care among PCPs who had postimplementation EMR training increased during the duration of the study [10]. The other 2 studies [16, 18], found that poor-quality vendor training was a barrier to physicians’ use of advanced EMR features. In one of the studies [16], in which a feedback survey was conducted, several physicians made negative comments regarding the quality of EMR vendor support and training. Furthermore, EMR vendor training was limited, and common support resources available were peer mentors and colleagues [16]. Another study [18],,found that physicians quickly forgot the content of training sessions provided by vendors if they were short and covered only technical functionalities. In addition, training material in these sessions was based on theory rather than practice and focused on basic functionalities that emphasized administrative benefits of EMRs rather than clinical ones. Moreover, the study [18] found that vendors were usually the source of information but that their availability was limited. These vendor based limitations biased physicians towards using only the basic functionalities, which they could easily recall when using the EMR. Interestingly, one study [7] found that practices that successfully used advanced EMR features dedicated time and resources to training and communication on how to use the advanced features (e.g., group training, procedural workflow manuals, 1-on-1 guidance).

### Coaching and peer mentoring

Three studies [16, 40, 44] found that support programs that involved coaching from consultants or peer mentoring facilitated the mature use of EMRs by physicians. One study [16] found that physicians who participated in a support program that involved coaching and peer mentoring reported that their meaningful use of EMR increased, which these physicians believed would positively impact their patients. In another study [44], consultants who used change management techniques (e.g., workflow analysis and corrections) to engage with physicians showed an improvement in physicians’ mature use of EMRs. Another study [40], conducted in the US, found that over half of PCPs who enrolled in a regional extension center (REC) program reported meaningful use of advanced EMR features. The RECs supported PCPs in achieving meaningful use of EMRs through education, technical assistance, and coaching.

### Sharing resources

Information sharing between practices and other healthcare organizations was highlighted in two studies [7, 39] as a key facilitator of the use of advanced EMR features. In one of the studies [39], PCPs who had shared technical support were more likely to have multifunctional health information technology capacity, electronically exchange patient information, and electronic patient access.

### Financial incentives

One study [39] found that physicians who received or were eligible for financial incentives were more likely to be able to electronically exchange patient information with physicians outside of their practice than those not eligible for incentives. However, the study found no association between incentives and multifunctional health information capacity or patient electronic access. Another study [7] found the costs of upgrading EMR systems to be a barrier in the use of advanced EMR features and highlighted that financial support was key to overcoming this challenge.

### Technical support

Study [7] highlighted that adequate technical support (e.g., vendor support, a health information technology department, or an in-house EMR “go-to person”) was a critical factor in the advanced use of EMRs because it supported the configuration of new EMR features and staff training.

### Resource availability

Study [18] reported that a further barrier to the mature use of EMRs was the paucity of sources of information about EMRs available to PCPs, since vendors were usually the main source of information but were not readily available.

#### Policy

Policies to increase EMR use are an important factor in EMR use, which was highlighted in only one of the studies. An international study [3] found that small practices in countries that had collaborative and regional policies to increase the spread and use of health information technology had EMR usage patterns of multifunctional capacity across four domains: the generation of patient information, the generation of patient registry information, order entry management, and decision support functionality.

## Discussion

Our systematic review found multiple interrelated factors that influenced the mature use of EMRs by PCPs. Five themes emerged across the 14 studies: technology, people, organization, resources, and policy. Concerns about an EMR system’s functionality, utility, ease of use, and technical reliability appeared to deter physicians from using advanced EMR features [7, 45]. As pointed out in one of the studies [43], physicians used particular EMR features more extensively when such features “fit” their main medical tasks. This key point regarding physicians perceiving a lack of fit between EMR systems and their values, priorities, and work practices was echoed in several studies [46–48]. This complements what was raised in a separate study [49], an evidence synthesis that concluded that the challenge of sustaining the use of technology in the healthcare field was due to the fact that many healthcare organizational processes (e.g., team-based care delivery, handovers) were immature, not standardized, and still evolving. This process immaturity makes it difficult for health information technology to support these processes [49].

Individual factors were the most common theme influencing PCPs’ mature use of EMRs. Gender appeared to affect the mature use of EMRs [10, 41]. There was mixed evidence regarding PCPs’ EMR experience and mature EMR use. Physicians who perceived that their EMR system was easy to use, reduced their cognitive load, improved communication, improved the quality of care, enhanced patient safety, improved the clinic’s workflow, and enhanced the efficiency of physicians made the widest-ranging use of their EMRs [41, 45]. A lack of physician awareness of EMR functionality and the lack of physician readiness to learn advanced features were issues emphasized in another study [17]. Physician motivation to advance their EMR use and user satisfaction were both found to be critical factors associated with mature EMR use [16, 18, 42, 44]. Furthermore, limited physician willingness to learn more advanced EMR features [16, 18], the habitual use of performing clinical tasks using basic functions [18], and patient concerns about the impersonality of the physician entering data during a consultation [7], were factors that were also found to prevent the maturation of EMR use.

Organizational factors were another theme that emerged from the studies. We found that practices affiliated with an integrated delivery system were associated with physicians who were advanced EMR users [37, 39, 41]. However, mixed reviews were found regarding practice size and physicians’ mature EMR use [40, 41], where larger practices achieved more advanced EMR use compared to solo practices [3, 7, 39]. However, as pointed out by Schoen et al. [3], even small practices can achieve mature EMR use if given health policy support and appropriate incentives. Organizational objectives were also found to influence PCPs’ mature use of EMRs. One study [18] reported that when a clinic’s objectives were overshadowed by operational objectives (e.g., operational efficiency), the effort by PCPs to achieve mature use of EMRs was thwarted. Another study [7] highlighted the importance of using team-based methods (e.g., giving responsibility to nonphysician staff to collect and enter patient data into EMRs) and transition planning (e.g., developing new processes and workflow procedures when implementing advanced features) to facilitate the use of advanced EMR features by PCPs.

Another dominant theme in this review was resources such as peer mentors or colleagues, coaching consultants, technical support, training, sharing resources with other healthcare organizations, and financial incentives, all of which had a favourable impact on PCPs’ use of advanced EMR features [7, 10, 16, 39, 40, 44]. On the other hand, limited and poor training by vendors was found to be a barrier to the use of advanced EMR features by PCPs [16, 18]. We also found that efforts made to provide adequate training both during and post EMR implementation that focused on clinical rather than administrative benefits had a favourable impact on PCPs’ mature use of the system [7, 10, 16, 18]. Finally, concerns about limited resources for learning more about EMRs and their features were also reported as a barrier [18].

Our findings highlight the key factors that affect the mature use of EMRs by PCPs. To enhance the mature use of EMRs we suggest focusing on the following factors. First, future EMR implementation programs must provide an opportunity for end-users to play an active role in the design process from the outset, which may help to address the barrier of a lack of fit between EMR features and clinicians’ priorities. Moreover, this approach could alleviate the functionality barriers of EMR systems and physicians’ concerns regarding the impersonality of their interaction with patients when using an EMR during a consultation. Second, providing adequate training that meets the needs of the end user could limit the habitual use of only basic EMR features and facilitate physician awareness of advanced EMR functionality and physician readiness to use advanced features. OntarioMD has developed a peer leader program that connects practices with clinicians who are superusers to support a practice’s mature use of EMRs [50]. Finally, initiatives that encourage physicians to join an integrated delivery system should be implemented, since out study revealed that this type of practice influences the mature use of EMRs by clinicians. One such initiative is Ontario’s funding of family health teams (a team-based model) [51] to assist with EMR use [52, 53]. Financial incentives could be one strategy to promote integrated delivery system practices, which may also encourage the use of EMR supported team-based models. Moreover, the implementation of collaborative and regional policies that provide technical and financial incentives (e.g., grants or reimbursements) could influence clinics to orient their organizational objectives towards maturing physicians’ EMR use rather than being business-oriented. In addition, policies that assist clinics in transition planning to develop new processes and workflow procedures when implementing advanced EMR features would be advantageous. Thus, collaborative and regional policies that support increased EMR use are another strategy to further the mature use of EMRs by PCPs. This was the case in the United States with the introduction of the “meaningful use” guidelines where empirical evidence shows positive results on EMR usage among physicians [54]. Last, the factors identified in this study operate interdependently and should not be taken into account in static isolation; how these key factors change over time should be considered when observing changes in the level of mature EMR use [55, 56]. Therefore, a key recommendation is to perform a longitudinal analysis on primary care practices to measure the progress of these maturity factors over time. This will allow monitoring of the progress of maturity of EMR use by primary care physicians while also taking into account the different maturity stages of an individual user.

### Limitations and strengths

One strength of our review is that the systematic review methodology we used was appraised using the AMSTAR instrument and rated as meeting 14 out of 16 criteria [36, 57]. In terms of the 2 missing items, they were not applicable because we did not conduct a meta-analysis (methods used to combine the findings of appropriate studies), and we did not assess publication bias. A limitation of our review is the small number of studies identified after exclusion (i.e., 14 eligible studies). One of the reasons for this low count is that our review focused on PCPs, which may limit the generalizability of our findings to other health care professionals and beyond primary healthcare [17, 58]. However, as highlighted in previous studies [15, 59], the literature in this field may be poorly referenced within bibliographic databases because terminology is not standardized, and there is no taxonomic consensus related to health information technologies [60]. This may also explain the limited number of eligible studies retrieved. Additionally, most studies included were conducted in North America, which is not surprising since the concept of meaningful use originated in the US and was developed vis-à-vis mature EMR use in Canada. Furthermore, the term “mature EMR use” was not consistent among studies, which may also have contributed to the limited number of studies that were included. There may have been studies on EMRs and primary care that are not included in this review because they refer to physician factors or contextual issues as something other than maturity. Thus, a key finding from our study is the need for a common terminology to define EMR maturity.

## Conclusions

The evidence provided by the studies in this review demonstrates that there are several linked factors that influence the mature use of EMRs by PCPs that could usefully inform future initiatives to sustain health information technologies within a primary care setting. Policymakers and vendors need to be aware that a primary care setting is a complex, dynamic environment, and initiating strategies that are informed by these factors have the potential to support the mature use of EMR by PCPs.

## Supplementary Information

**Additional file 1**. Confidence assessments. Assessment of confidence in synthesis findings.

## Data Availability

All data generated or analyzed during this study are available in this published article and its supplementary information file.

## Abbreviations

EMR: Electronic Medical Record
PCP: Primary Care Physician
PRISMA: Preferred Reporting Items for Systematic Reviews and Meta-Analyses
PHO MetaQAT: Public Health Ontario Meta quality appraisal tool
